# Acute myocardial infarction complicated by cardiogenic shock in Ukraine: multicentre registry analysis 2021–2022

**DOI:** 10.3389/fcvm.2024.1377969

**Published:** 2024-03-28

**Authors:** Anton O. Bilchenko, Olga V. Gritsenko, Volodymir O. Kolisnyk, Oleg I. Rafalyuk, Andrii V. Pyzhevskii, Yaroslav V. Myzak, Dmytro I. Besh, Victor M. Salo, Sergii O. Chaichuk, Mykhailo O. Lehoida, Ihor V. Danylchuk, Ihor V. Polivenok

**Affiliations:** ^1^ Department of Prevention and Treatment of Emergency Conditions, L.T. Malaya Therapy National Institute of the National Academy of Medical Sciences of Ukraine, Kharkiv, Ukraine; ^2^ Department of Interventional Cardiology, V.T. Zaitcev Institute of General and Urgent Surgery of the National Academy of Medical Sciences of Ukraine, Kharkiv, Ukraine; ^3^ Department of Interventional Cardiology, Odesa Regional Hospital, Odesa, Ukraine; ^4^ Department of Interventional Radiology, Lviv Regional Clinical Treatment and Diagnostic Cardiology Center, Lviv, Ukraine; ^5^ Department of Interventional Radiology, 1st Territorial Medical Union, Lviv, Ukraine; ^6^ Department of Family Medicine, Danylo Halytsky National Medical University, Lviv, Ukraine; ^7^ Department of Interventional Cardiology, Oleksandrivska Clinical Hospital, Kyiv, Ukraine; ^8^ Department of Cardiology, Vinnytsia Regional Clinical Treatment and Diagnostic Center of Cardiovascular Pathology, Vinnytsia, Ukraine; ^9^ Department of Therapy No 1, Kharkiv National Medical University, Kharkiv, Ukraine

**Keywords:** cardiogenic shock, acute myocardial infarction, mortality risk factors, clinical outcomes, mechanical circulatory support

## Abstract

**Background:**

Data on the results and management strategies in patients with acute myocardial infarction complicated by cardiogenic shock (AMI-CS) in the Low and Lower-Middle Income Countries (LLMICs) are limited. This lack of understanding of the situation partially hinders the development of effective cardiogenic shock treatment programs in this part of the world.

**Materials and methods:**

The Ukrainian Multicentre Cardiogenic Shock Registry was analyzed, covering patient data from 2021 to 2022 in 6 major Ukrainian reperfusion centres from different parts of the country. Analysis was focusing on outcomes, therapeutic modalities and mortality predictors in AMI-CS patients.

**Results:**

We analyzed data from 221 consecutive patients with CS from 6 hospitals across Ukraine. The causes of CS were ST-elevated myocardial infarction (85.1%), non-ST-elevated myocardial infarction (5.9%), decompensated chronic heart failure (7.7%) and arrhythmia (1.3%), with a total in-hospital mortality rate for CS of 57.1%. The prevalence of CS was 6.3% of all AMI with reperfusion rate of 90.5% for AMI-CS. In 23.5% of cases, CS developed in the hospital after admission. Mechanical circulatory support (MCS) utilization was 19.9% using intra-aortic balloon pump alone. Left main stem occlusion, reperfusion deterioration, Charlson Comorbidity Index >4, and cardiac arrest were found to be independent predictors for hospital mortality in AMI-СS.

**Conclusions:**

Despite the wide adoption of primary percutaneous coronary intervention as the main reperfusion strategy for AMI, СS remains a significant problem in LLMICs, associated with high in-hospital mortality. There is an unmet need for the development and implementation of a nationwide protocol for CS management and the creation of reference CS centers based on the country-wide reperfusion network, equipped with modern technologies for MCS.

## Graphical Abstract

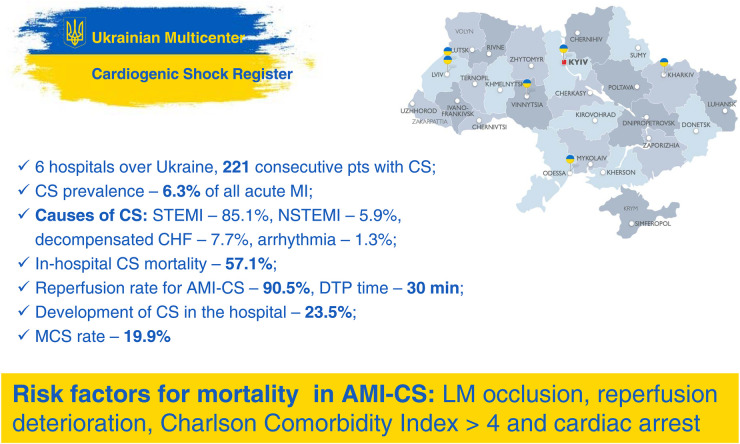

CS, cardiogenic shock; AMI-CS, acute myocardial infarction complicated by cardiogenic shock; STEMI, ST-elevation myocardial infarction; NSTEMI, non-ST-elevation myocardial infarction; CHF, chronic heart failure; DTP, door-to-procedure; MCS, mechanical circulatory support; LM, left main stem; PCI, percutaneous coronary intervention; CABG, coronary artery bypass grafting.

## 1 Introduction

The widespread dissemination of reperfusion therapy for acute myocardial infarction (AMI) based on the primary percutaneous coronary intervention (pPCI) and the development of reperfusion networks has led to significant improvement in survival and decrease in complications rate in AMI (1). However, among patients with acute myocardial infarction complicated by cardiogenic shock (AMI-CS) hospital mortality has not changed significantly over the past 20 years, despite progress in reperfusion therapy and mechanical circulatory support (MCS), and remains about 30%–50% (2–4). Available data demonstrate that early and effective reperfusion is the key factor in reducing mortality in AMI-CS patients (5). However, the effectiveness of reperfusion therapy declines with the prolongation of total myocardial ischemic time (6, 7). Moreover, rapid reperfusion may cause additional myocardial damage by itself, which exacerbates the course of cardiogenic shock (CS) (8, 9).

In addition, obtaining of strong evidence for the most effective CS management, particularly for MCS, is complicated by clinical polymorphism of CS and the lack of tools for patients' stratification according to the shock severity in the majority of previous studies. The new clinical classification of CS, proposed by Society for Cardiovascular Angiography and Interventions (SCAI) in 2018, has been designed to solve this problem (10). It should also be noted that there is a clear shortage of large prospective randomized trials of CS secondary to ethical and methodological challenges in the randomization of critical patients (5), thus retrospective registries still play a significant role in CS trials. The purpose of the study is to evaluate the incidence, risk factors, therapeutic options, and outcomes among patients with AMI-CS in the programs with limited access to the MCS.

## 2 Materials and methods

### 2.1 Description of the reperfusion centers and the registry

In 2020, a Ukrainian Multicentre Cardiogenic Shock Registry was launched. By 2021, the registry included 6 major Ukrainian reperfusion centres in Kharkiv, Kyiv, Vinnytsia, Lviv and Odesa, covered different parts of the country. All of the participating reperfusion centers are the parts of the hospitals with catheterization laboratories available 24/7 for AMI and other cardiac emergencies care. All hospitals have cardiac surgery on site and dedicated cardiac ICUs. For the MCS, intra-aortic balloon pump (IABP) and extracorporeal membrane oxygenation (ECMO) are available in 3 hospitals, percutaneous ventricular assist devices (pVADs) are not available in any hospital. An online registry was developed and launched by the team from Kharkiv reperfusion center. Responsible physician was designated in each of the participating hospitals who was instructed in the SCAI shock criteria and entering data into the registry. Patients with shock were included to the registry at each center at the discretion of the designated physician, with Zoom consultations provided when necessary. The data from these centres allow to generate and systematize information on CS based on a sufficiently wide sample of at least 7 million population. The participating centres represent the healthcare infrastructure in general, and patient population across all regions of Ukraine. All previous information on CS in Ukraine was not systematic or did not focus on this issue. This study assesses detailed information on each case of CS from 1 January 2021 to 24 February 2022. Data collection ceased on the latter date due to the commencement of the Russian military invasion, which imposed significant disruptions on our research activities. After the almost 2 years break due to the destruction of our activities by the war, we were finally able to resume the maintenance of the Ukrainian Cardiogenic Shock registry.

### 2.2 Study design and patient selection criteria

**Figure 1:**
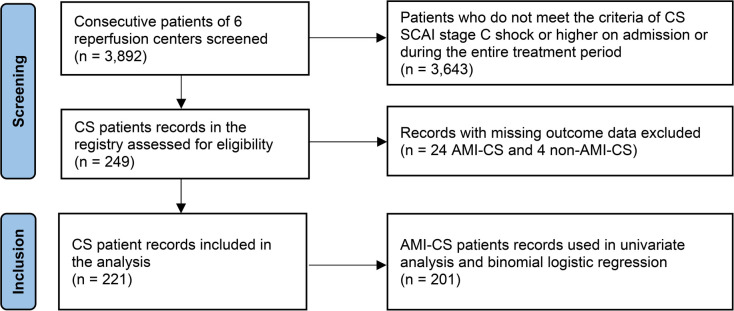
Study flowchart.

The study is a registry-based retrospective observational analysis of CS in Ukraine. Overall, 3,892 consecutive patients with acute cardiac conditions admitted to the reperfusion centers were screened. Vast majority of them (3,596 patients) had an AMI as a cardiac emergency. After review of the sources including medical records, discharge reports and local databases, 221 patients meeting SCAI criteria of CS (10) at least stage C either on admission or during hospitalization were finally selected for the analysis (Figure 1).

### 2.3 Ethical declaration

All patients enrolled to the study, or their relatives signed an informed consent about personal data use according to procedure approved by Ministry of Healthcare of Ukraine. The study was performed in accordance with World Medical Association Declaration of Helsinki 1964, amended in 2013, and approved by the local ethics committee.

### 2.4 Diagnosis of acute myocardial infarction and reperfusion strategy

The diagnosis of AMI, as well as the choice of reperfusion strategy for all patients treated in reperfusion centers, was determined by the current recommendations of European Society of Cardiology (ЕSC) (11, 12).

### 2.5 Percutaneous coronary interventions

In cases of acute coronary syndrome invasive coronary angiography was performed immediately after hospitalization. In most cases, myocardial revascularization in acute phase was limited by stenting of culprit lesion only. Manual thrombus aspiration was performed at the discretion of the catheterization laboratory team.

### 2.6 Concomitant medication and mechanical circulatory support

The routine initial medication followed ESC recommendations (11, 12) and included loading doses of aspirin with ticagrelor, prasugrel or clopidogrel, high doses of statins and unfractionated heparin 70 IU/kg during PCI unless contraindicated. All subsequent medical therapy dependent on clinical scenario and comorbidities. Except IABP any other types of MCS are either not available in Ukraine at all (e.g., pLVAD) or very limited mostly in centers with cardiac surgery on site [Veno-Arterial Extracorporeal Membrane Oxygenation (VA-ECMO)], which reflects the situation in a vast majority of Ukrainian reperfusion centers.

### 2.7 Clinical endpoints

In-hospital mortality was used as clinical endpoint.

### 2.8 Risk factors, concomitant and emergency conditions

Data on cardiovascular risk factors and concomitant diseases were obtained by review of primary medical records, interview of the patients or phone contact to the family physicians. Heart failure was diagnosed in accordance to the recommendations of the ESC (13, 14). The Charlson Comorbidity Index was used to assess the number and severity of comorbid conditions for each patient in a study (15). Reduced glomerular filtration rate (rGFR) was defined in our study as a GFR less than 60 milliliters per minute per 1.73 square meters (ml/min/1.73 m^2^). Cardiac arrest was diagnosed in the presence of at least one of the following conditions: asystole, electromechanical dissociation, ventricular fibrillation, or pulseless ventricular tachycardia. Reperfusion deterioration was defined as an additional hemodynamic compromise longer than 30 min after the opening of infarct-related artery (IRA), required additional therapeutic interventions and clinically manifested as the recurrent arrhythmia, systemic hypotension, pulmonary edema, or an increase in SCAI stage of CS by one step or more. The blood flow in the IRA after revascularization was assessed by TIMI score (16). Concomitant chronic total occlusion (CTO) of the coronary artery was diagnosed in the case of complete non-IRA arterial occlusion with or without angiographic collateral blood flow (17). The duration of total ischemic time was verified as the total time from the onset of symptoms to the beginning of PCI. Multivessel coronary disease was defined as documented angiographic stenoses >50% of the diameter of two or more coronary arteries. The usage of IABP, inotropic support and mechanical ventilation was determined by local hospital protocols, strongly recommended to be adjusted to the currently available recommendations (11).

### 2.9 Data representation

Data was collected using a range of qualitative and quantitative indicators. Qualitative data was categorized into different groups and represented as percentages to allow for easy comparison. For the quantitative data, a 95% confidence interval (CI) was used.

### 2.10 Statistical analysis

Statistical analysis was performed using the SPSS for Mac software package, version 26 (IBM, Chicago, USA) and Jamovi Desktop 2.3.18. Categorical variables were presented as numbers and percentage, continuous ones—as the median and interquartile range (IQR). To assess the differences between subgroups, the *U* Mann–Whitney test and Fisher's exact test were used. To identify the risk factors for hospital mortality we used univariate and multivariate analysis, followed by calculating the odds ratio (OR) and 95% CI for each of the factors. Binomial logistic regression was employed to estimate the influence of various factors on a hospital mortality. Each factor was evaluated based on estimates, standard error, z-score, *p*-value, odds ratio, and a 95% confidence interval. The performance of the regression model was tested using a Receiver Operating Characteristic (ROC) curve. All statistical tests were two-sided, with *p*-values less than 0.05 considered statistically significant.

## 3 Results

A total of 221 patients with CS were included in the analysis. In 85.1% of cases the cause of CS was STEMI, in 5.9%—NSTEMI, in 7.7%—decompensated CHF and in 1.3% it was arrhythmia.

Baseline clinical characteristics of the patients are represented in Table 1. In general, patients with non-AMI-CS were more likely to have a history of MI and more comorbidities (high Charlson Comorbidity Index, reduced renal function, and CHF) compare with AMI-CS patients.

**Table 1:** Characteristics of the patients at baseline.

Characteristic	Total	Non-AMI-CS	AMI-CS	*p* *
	(*n*=221)	(*n*=20)	(*n*=201)	
Age—yr, Mdn (IQR)	69 (60.5–77)	73 (64–82)	68 (60–77)	ns
Male sex—no. (%)	130 (58.8)	12 (60)	118 (58.7)	ns
CS causes—no. (%)				
NSTEMI	13 (5.9)	–	13 (6.4)	
STEMI	188 (85.1)	–	188 (93.6)	
Decompensated HF	17 (7.7)	17 (85)	–	
Others	3 (1.3)	3 (15)	–	
History of MI—no. (%)	75 (33.9)	18 (90)	57 (28.3)	0.0001
History of PCI/CABG—no. (%)	16 (7.2)	3 (15)	13 (6.4)	ns
Hypertension—no. (%)	184 (83.3)	18 (90)	166 (82.5)	ns
CTO—no. (%)	74 (33.5)	4 (20)	70 (34.8)	ns
MVD—no. (%)	138 (62.4)	12 (60)	126 (62.7)	ns
Charlson comorbidity index (*n*=220)				
<4	50 (22.6)	1 (5)	49 (24.4)	ns
4–7	130 (58.8)	11 (55)	119 (59.2)	ns
>7	40 (18.1)	8 (40)	32 (15.9)	0.014
Comorbidities—no. (%)				
rGFR	131 (59.3)	16 (80)	115 (57.2)	0.057
DM	69 (31.2)	9 (45)	60 (29.8)	ns
CHF	87 (39.3)	18 (90)	69 (34.3)	0.0001
Total ischemic time—h, Mdn (IQR)			5 (3–9)	
DTP time—min, Mdn (IQR)			30 (20–45)	

AMI-CS, acute myocardial infarction complicated by cardiogenic shock; Mdn, median; IQR, 1 and 3 interquartile range; STEMI, ST-elevation myocardial infarction; NSTEMI, non-ST-elevation myocardial infarction; HF, heart failure; MI, myocardial infarction; PCI, percutaneous coronary intervention; CABG, coronary artery bypass grafting; CTO, chronic total occlusion; MVD, multivessel coronary disease; rGFR, reduced glomerular filtration rate; DM, diabetes mellitus; CHF, chronic heart failure; DTP, door-to-procedure.

* *p*—difference between Non-AMI- and AMI-CS subgroups.

Among 3,596 patients with AMI, CS developed in 225 (6.3%), 24 of which were excluded from the analysis due to missed outcome data. Out of 201 analyzed AMI-CS cases, 93.6% was a result of STEMI, and 6.4% of NSTEMI. 40.3% of AMI-CS patients experienced at least one episode of circulatory arrest, which occurred before (21.5%) or after (18.8%) PCI. The dominant infarct-related artery (IRA) was the left anterior descending (LAD) one in 48.9% of the cases. Additionally, 62.7% and 34.8% of patients had multivessel disease, and CTO respectively (Tables 1, 2).

**Table 2:** AMI-CS management characteristics.

Characteristic^a^	AMI-CS
	no (%)
Revascularization (*n*=201)	182 (90.5)
PCI	180 (89.5)
CABG	2 (1)
Infarct-related artery (*n*=190)	
LM	17 (8.9)
LAD	93 (48.9)
RCA	59 (31.1)
Cx	21 (11.1)
Pre-PCI TIMI flow (*n*=180)	
0–1	163 (90.6)
2–3	17 (9.4)
Post-PCI TIMI flow (*n*=181)	
0–1	43 (23.8)
2–3	138 (76.2)
Reperfusion deterioration (*n*=200)	129 (64.5)
Development of shock after admission (*n*=200)	47 (23.5)
2 or more inotropes/vasopressors (*n*=201)	88 (43.8)
Intra-aortic balloon pump (*n*=201)	40 (19.9)
Before PCI	26 (12.9)
After PCI	14 (7)
Mechanical ventilation (*n*=193)	79 (40.9)
Before PCI	36 (18.7)
After PCI	43 (22.2)
Cardiac arrest (*n*=186)	75 (40.3)
Before PCI	40 (21.5)
After PCI	35 (18.8)

PCI, percutaneous coronary intervention; CABG, coronary artery bypass grafting; LM, left main stem; LAD, left anterior descending artery; RCA, right coronary artery; Cx, circumflex artery; TIMI, thrombolysis in myocardial infarction flow grade.

^a^ —data represented in numbers (%) of available records.

In AMI-CS patients emergent revascularization was performed in 90.5% of cases, mostly by PCI (89.5%) with few CABG (1%). After PCI was performed on the IRA, 35.8% of the patients experienced severe reperfusion disorders followed by the progression of the CS, and 18.8% experienced cardiac arrest after PCI. Failure of immediate coronary flow restoration with a post-PCI TIMI 0–1 flow was observed in 23.8% of the patients (Table 2). Median total ischemic time time for STEMI patients was 4 h (IQR, 3–9), and door-to-procedure time was 30 min (IQR, 20–45). In 23.5% of cases CS developed in the hospital after admission.

The following main therapeutic modalities—more than one inotrope/vasopressor, mechanical ventilation, and IABP—were used in 43.8%, 40.9%, and 19.9% of patients respectively (Table 2).

Overall, in-hospital mortality rate was 57.5%. There was a trend toward higher mortality in CS without AMI compared to AMI-CS—80% vs. 55.7%, without reaching a statistically significant difference (*p* = 0.0552).

**Figure 2:**
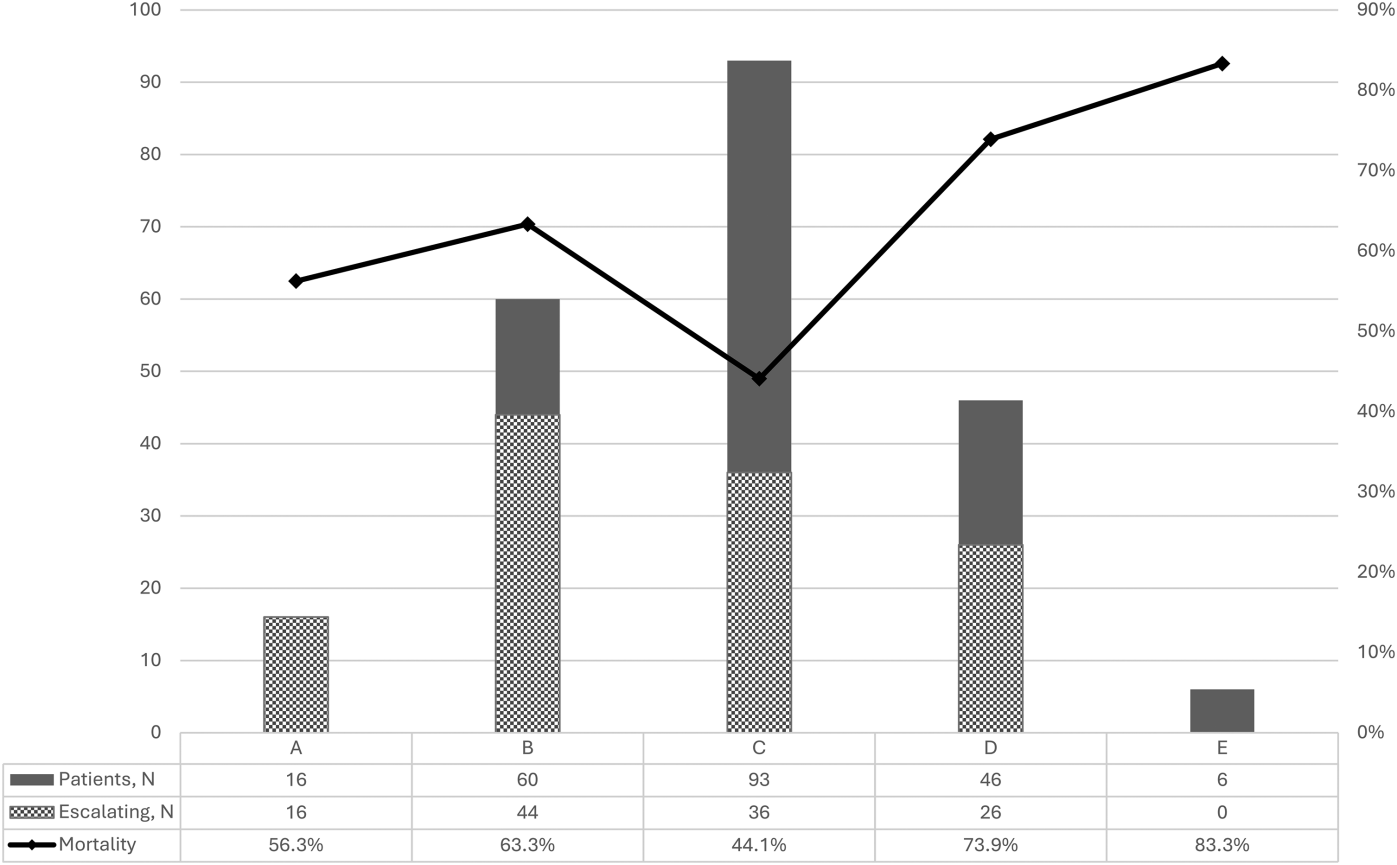
Number of patients, proportion of shock escalation and mortality according to SCAI stages of cardiogenic shock.

The relationship between SCAI stages on admission, escalation of shock and mortality is presented in Figure 2. Most patients were admitted in stage C (42.1%). The highest mortality rate was observed in stage E—83.3%, and the lowest one in stage C—44.1%. Patients admitted without shock who developed shock in hospital (stage A) and stage B patients had a mortality rate of 56.3% and 63.3%, respectively. The progression of shock during the hospital stay was 73.3% for stage B, 38.7% for C and 56.5% for D. Obviously, all patients with stage A in our registry progressed, and stage E is the last one, so the percent of escalation in these stages (100 and 0) is irrelevant.

**Figure 3:**
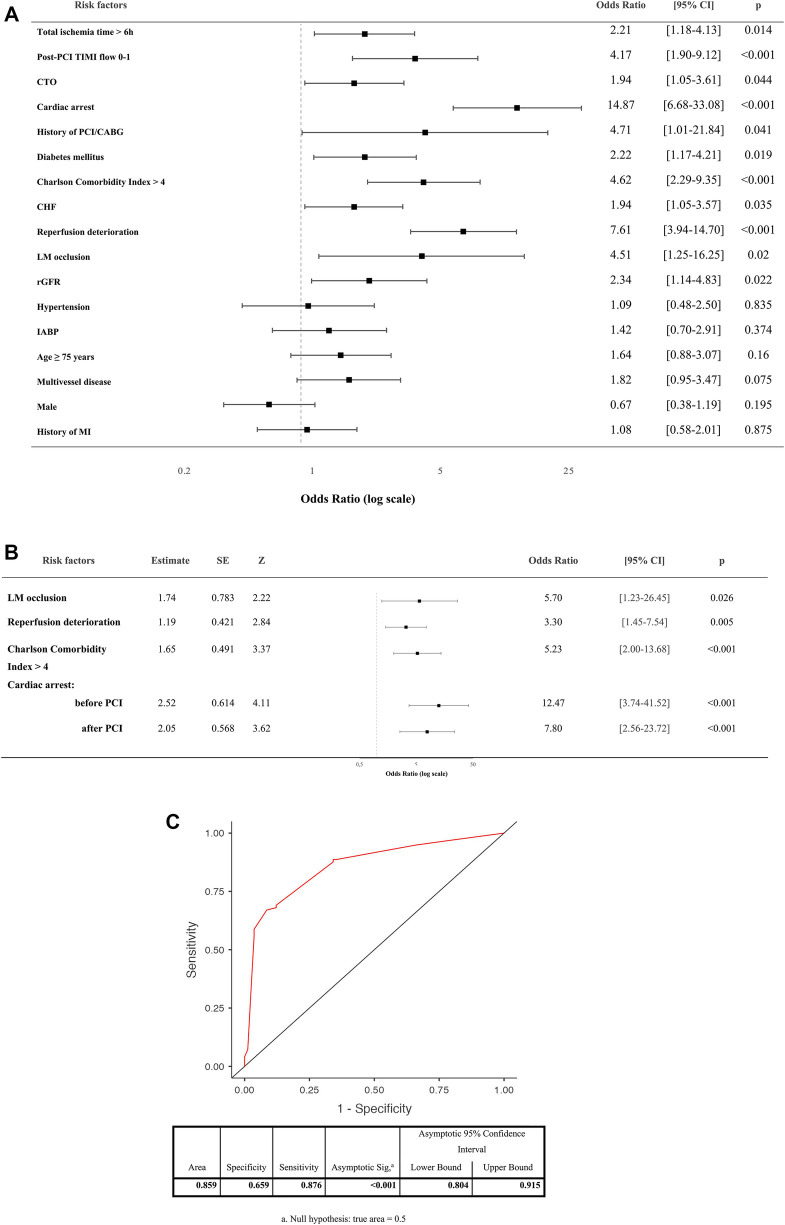
(**A**) Univariant analysis of risk factors for hospital mortality in AMI-CS patients. (**B**) Independent predictors for hospital mortality of CS patients by binomial logistic regression (coefficient-outcome model). (**C**) Receiver operating characteristic (ROC) curve of the logistic regression model. PCI, percutaneous coronary intervention; TIMI, thrombolysis in myocardial infarction flow grade; CABG, coronary artery bypass grafting; LM, left main stem; CTO, chronic total occlusion; rGFR, reduced glomerular filtration rate; MI, myocardial infarction; CHF, chronic heart failure; IABP, intra-aortic balloon pump.

Results of univariant analysis of risk factors for hospital mortality in AMI-CS patients is presented in Figure 3A.

Binomial logistic regression has yielded that the four factors (LM occlusion, deterioration after reperfusion, Charlson Comorbidity Index >4 and cardiac arrest) have remained independent predictors for hospital mortality (Figure 3B). A model with independent risk factors derived from multivariate regression showed high sensitivity and specificity (Figure 3C).

We evaluated the mean scores of different groups based on the outcomes. The negative outcome group, characterized by death, had a longer total ischemic time time with median (Mdn) of 6 h compared to the positive outcome group, characterized by survival, with a median of 4 h (*U* = 5,884.5, *p* < 0.001). Door-to-procedure time was also longer on average in the negative outcome group (Mdn—32.5 min) compared to the positive outcome group (Mdn—30 min) (*U* = 3,852, *p* = 0.002). Glomerular filtration rate was significantly lower in patients with a negative outcome (Mdn—40.7 ml/min/1.73 m^2^), compared to those with a positive outcome (Mdn—47.5 ml/min/1.73 m^2^) (*U* = 2,931, *p* = 0.006).

## 4 Discussion

In our study the incidence of AMI-associated CS was 6.3%, which was lower compared to large registries, where the CS rate was 7.9%–8.9% amid patients with STEMI (3, 18). These findings can relate to the fact that a slightly different CS criteria are used in different registries. The introduction of the recent SCAI classification into clinical practice could contribute to the unification of approaches to CS and the use of a common language for all stakeholders (10). In addition, we observed that since the introduction of the reperfusion network in Ukraine in 2016, the frequency of CS admitted to reperfusion centers continues to increase as the network improves. In the future, we should expect an increase in patients with CS in Ukraine up to the rate comparable to Western countries.

Hospital mortality for CS in our series was 57.1%, which is comparable to the data of a London registry that showed mortality rate of 45%–70% among 1,890 patients with CS with no tendency to decline over 9 years (3). In another large American registry, there was a significant decrease in hospital mortality for CS from 44.6% in 2003 to 33.8% in 2010 (18). Whether these differences are associated with different treatment strategies or are the result of the different criteria for CS remains unclear. A more recent registry series have shown an increase in survival among patients with CS up to 63%–82% when using dedicated teams, an early left ventricular unloading strategy and advanced MCS (pLVAD, MCS escalation) (19–21).

In our study, patients with non-AMI-CS tended to have higher in-hospital mortality rate compared to patients with AMI-CS (80% vs. 55.7%). This is inconsistent with previous data from the observational CardShock study (22), which showed a higher survival rate for patients with non-ACS etiology of CS. This difference can be explained by the facts that in our study (i) there were few patients with non-AMI-CS, and (ii) IABP, which is considered the most effective, especially for non-ACS-CS, was only used in 3 out of 20 patients (15%) in non-AMI-CS subgroup.

The highest mortality in our registry was among the most severe stages of shock—D and E (73.9% and 83.3% respectively), which is quite explainable by the severity of shock, and the very low level of MCS in real practice in Ukraine. However, a more interesting finding for understanding of the shock management is that a vast majority of patients with stage B (73.3%) experienced further shock escalation, and the mortality rate in this group was significantly higher than among patients who presented with stage C, where only 38.7% of patients experienced this escalation of the shock. Even patients who developed shock after hospital admission (stage A) had a higher mortality rate compared to the patients with stage C on admission. These findings are entirely consistent with those of the Cardiogenic Shock Working Group registry, which found the same trends in an analysis of 3,455 patients with CS (23). The obtained data suggest that patients in the “pre-shock” (stage B, sometimes even A) are often underestimated upon hospital admission in terms of risk assessment and do not receive adequate therapy on time. While patients with “classic” shock upon admission usually receive proper monitoring and treatment from the very first minute. Shifting the focus of shock management toward early detection, early invasive monitoring, and more aggressive management of pre-shock patients may be a reasonable strategy to improve survival.

Overall, the incidence of MCS, exclusively in the form of IABP, in our registry was 19.9%, which is significantly lower than was demonstrated in a recent large US registry, where the frequency of MCS-assisted early PCI in patients with AMI-CABG was about 50% (4). The use of IABP in our study had no impact on mortality. Data from randomized trials and meta-analysis (24–29) confirmed the lack of IABP's effect on the survival of patients with CS, leading to downgrading of the indication for the routine use of IABP in CS to class III (11). Nevertheless, a recent registry series has shown improved outcomes with IABP (30), and IABP remains the most common MCS modality in the US (4, 31).

In addition, it remains unclear whether an early use of IABP has potential benefits in AMI patients with the risk of developing CS (e.g., SCAI stage A). As for other MCS options, such as pLVAD or VA-ECMO, these technologies are either not available in Ukraine (Impella, TandemHeart etc.), or are limited to a small number of centers with advanced cardiac surgery (VA-ECMO) and had no impact on real clinical practice.

In general, a key component of the treatment of CS is the shock team, which uses the local protocols based on early identification, advanced hemodynamic monitoring and MCS escalation/de-escalation strategies driven by such monitoring.

Management of AMI-CS poses unique challenges, particularly LLMICs. In such countries, resource constraints and limited access to advanced medical interventions often impact patient care and outcomes. Furthermore, disparities in healthcare personnel training and facility distribution can compromise the standard of care. As a LLMIC, Ukraine embodies these challenges. Thus, while the existing body of knowledge about AMI-CS risk factors and management is expanding globally, it is crucial to apply this knowledge within the specific context of Ukraine's healthcare landscape. Consequently, our study aimed to examine these variables, building upon the existing knowledge within the context of AMI-CS in Ukraine.

Previous studies, most of which, if not all, carried out in high-income countries, have identified the following risk factors for hospital mortality in CS: etiology of acute coronary syndrome, older age, history of AMI or coronary artery bypass grafting (CABG), ischemic brain damage, reduced LVEF, impaired right ventricular function, mitral regurgitation, decreased LV stroke work and cardiac power output, systolic blood pressure, the number of vasopressors to support hemodynamics, serum lactate level, systemic inflammatory response syndrome, and TIMI flow in IRA (22, 32–36).

In our study univariate analysis has revealed that TIMI 0–1 after reperfusion, chronic total occlusion, previous history of PCI/CABG, diabetes mellitus, Charlson Comorbidity Index >4, chronic heart failure, reduced GFR, LM occlusion, total ischemic time >6 h, deterioration after reperfusion and cardiac arrest were the risk factors for hospital death in patients with AMI-CS. However, LM occlusion, deterioration after reperfusion, Charlson Comorbidity Index >4 and cardiac arrest were independent predictors of hospital mortality related to AMI-CS.

## 5 Conclusion

Despite the wide adoption of primary PCI as a main reperfusion strategy, СS remains a significant challenge for the LLMIC healthcare system, associated with unacceptably high in-hospital mortality and a substantial burden on a resource-limited system. There is an unmet need to develop and implement a nationwide CS management protocol based on early identification, advanced hemodynamic monitoring and MCS escalation/de-escalation capability, to improve patient survival. We see a reliable solution in the creation of reference CS centers based on a reperfusion network in Ukraine, equipped with modern technologies for mechanical circulatory support.

## 6 Study limitations

This study has several limitations, some of which are inherent to the analysis of a web-based multicenter registry. (i) The accuracy of diagnosis and the documentation of complications may have varied among different healthcare facilities. (ii) The study may not have adequately accounted for variations in treatment protocols and healthcare provider practices across different centres. This might impact the generalizability of the results. (iii) As this is a registry analysis, the lack of randomization might lead to selection bias and confounding, potentially affecting the interpretation of the results. (iv) The study did not include long-term follow-up data that could provide important insights into the development and outcomes of patients with AMI-CS.

## Data Availability

The raw data supporting the conclusions of this article will be made available by the authors, without undue reservation.
